# Acute Hemophagocytic Lymphohistiocytosis Due to Tularemia

**DOI:** 10.7759/cureus.69294

**Published:** 2024-09-12

**Authors:** Niravkumar K Brahmbhatt, Amber Pinson-Hilkemeyer, Saransh Narang, Tirth M Patel, Deesha Bhojwani, Sachin M Patil, William Roland

**Affiliations:** 1 Internal Medicine, University of Missouri Health Care, Columbia, USA; 2 Internal Medicine, University of Missouri Healthcare, Columbia, USA; 3 Medicine, Medical College of Baroda, Baroda, IND; 4 Medicine, Bukovinian State Medical University, Chernivtsi, UKR; 5 Internal Medicine/Infectious Diseases, University of Missouri Healthcare, Columbia, USA; 6 Internal Medicine/Infectious Diseases, Critical Care, Pulmonary, Universal Health Services (UHS) Southwest Healthcare, Temecula, USA

**Keywords:** francisella tularensis, hemophagocytic lymphohistiocytosis (hlh), immune, septic shock, tularemia

## Abstract

Hemophagocytic lymphohistiocytosis (HLH) is a rare, life-threatening disorder marked by excessive immune system activation. Diagnosing HLH is particularly difficult due to its diverse etiology and similar clinical presentation. Patients with HLH often require intensive critical care. The condition is divided into HLH syndrome (genetic defects) and HLH disease, and both can be triggered by factors such as infections, autoimmune diseases, or malignancies. We present here the first documented case of acute HLH secondary to tularemia with septic shock in a 62-year-old female. Initially suspected due to malignancy-induced acute HLH, she was treated with etoposide and dexamethasone and was immediately transitioned to antimicrobials once she was confirmed positive for *Francisella tularemia* on blood cultures at the state microbiology laboratory. She had a prolonged clinical course of 31 days, followed by an excellent recovery after treatment with antimicrobials. This case highlights the importance of recognizing tularemia as a potential cause of HLH. Identifying and addressing the causative agent promptly is crucial for effective management and treatment of HLH.

## Introduction

Hemophagocytic lymphohistiocytosis (HLH) is an uncommon, ineffective, aberrant, dysregulated hyperimmune response. It is a life-threatening condition characterized by a hyperinflammatory state resulting in a cytokine storm, which causes organ dysfunction and an increase in mortality and morbidity [1]. HLH can be due to either genetic or acquired causes at any age. Acquired instigators include infections, malignancy, autoimmune diseases, immunosuppressants, and acquired immune defects. Two nationwide registries on adult HLH revealed infections as the primary cause among adults [2,3]. As the age increased, there was a greater predilection toward malignancy and other acquired etiology for HLH [2]. Among the infectious causes, tick-borne diseases (TBDs) are a rare cause of HLH. Tularemia, caused by the bacterium *Francisella tularensis* (*F. tularensis*), is a highly contagious zoonotic disease that typically manifests as an ulceroglandular disease. To date, on review of medical literature via PubMed, there have been no documented cases of HLH secondary to tularemia.

## Case presentation

A 63-year-old female with a prior medical history of hyperlipidemia, depression, and morbid obesity presented to an outside hospital (OSH) emergency department (ED) with fever, chills, dyspnea, nausea, vomiting, diarrhea, lethargy, and myalgia gradually worsening over the last four to five days. Approximately a week prior to the presentation, she admitted to a bug bite over the left thigh, followed by left groin swelling in three to four days, along with myalgia. Clinical examination at the ED revealed a respiratory rate of 18/minute, borderline low blood pressure of 95/62 mmHg with a normal oxygen saturation. Bilateral lung faint inspiratory crackles and tenderness in the left groin were noted. Lab findings disclosed leukopenia, thrombocytopenia, hyponatremia, hypokalemia, transaminitis, lactic acidosis, elevated creatinine, D-dimer, procalcitonin, LDH, and pro-BNP (Table 1).

**Table 1:** Inpatient laboratory results in a snapshot NP PCR: nasopharyneal polymerase chain reaction

Laboratory tests	Normal values	Day 1	Day 3	Day 4 - 7	Day 8	Discharge
1) White cell count	4,000–11,000/mL	3,000	2,530	14,300	3,190	17,320
2) Red cell count	4.3–5.6 million/mL	4.62	4.37	3.65	3.25	2.7
3) Hemoglobin	14–16 gm/dL	13.3	12.1	9.2	8.8	7.8
4) Neutrophil%	42.5–72%	90.6%	83%	93.4%	91%	76.8%
5) Lymphocyte%	21–51%	4.7%	14.6%	4.4%	8%	7.4%
6) Platelets	130,000–400,000/mL	99,000	50,000	81,000	17,000	124,000
7) Creatinine	0.5–1 mg/dL	4.2	7.1	2.3	2.3	1.69
8) Aspartate-aminotransferase (AST)	<32 U/L	134	382	657	253	52
9) Alanine-aminotransferase (ALT)	<35 U/L	49	87	120	100	138
10) Total bilirubin	<1.6 mg/dL	1.2	4.3	5.6	6.1	1.03
11) International normalized ratio (INR)	0.8–1.2		1.6			1.1
12) C-reactive protein (CRP)	<1 mg/dL	44.71			3.56	
13) Coronavirus disease-2019 NP PCR	Negative	Negative				
14) D-dimer	<500 ng/mL	> 8,000				
15) Erythrocyte sedimentation rate (ESR)	00–30 mm/Hr	15			1	
16) Ferritin	11.1–264 ng/mL		> 50,000	15,200	8,850	10,160
17) Fibrinogen	169–444 mg/dL				60	
18) Soluble interleukin 2 receptor (sIL-2R)	175.3–858.2 pg/mL		12,168		2381.6	
19) Lactic acid	0.5–2.2 mmol/L	3.3	4.9	3.6	2.1	
20) Lactate dehydrogenase (LDH)	313–618 U/L	2293		9967	4127	
21) NT-pro BNP (N-terminal pro-brain natriuretic peptide)	<300 pg/mL	3450				
22) Natural killer cell functional assay	>1				0.0	19.3
23) Procalcitonin (PCT)	<0.10 ng/mL	38.61		4.54	1.96	
24) Triglycerides	<150 mg/dL				1104	
25)HIV serology [Human immunodeficiency virus]	Negative	Negative				

Viral serology for coronavirus 2019 and human immunodeficiency virus returned negative. Blood cultures, stool studies, pathogen panel, and TBD serologies were sent. She was initiated on intravenous (IV) ceftriaxone and given a liter (L) of normal saline (NS). She became hypotensive (71/60 mmHg) and improved with 2 L of NS, and antimicrobials were escalated to vancomycin and zosyn.

**Figure 1:**
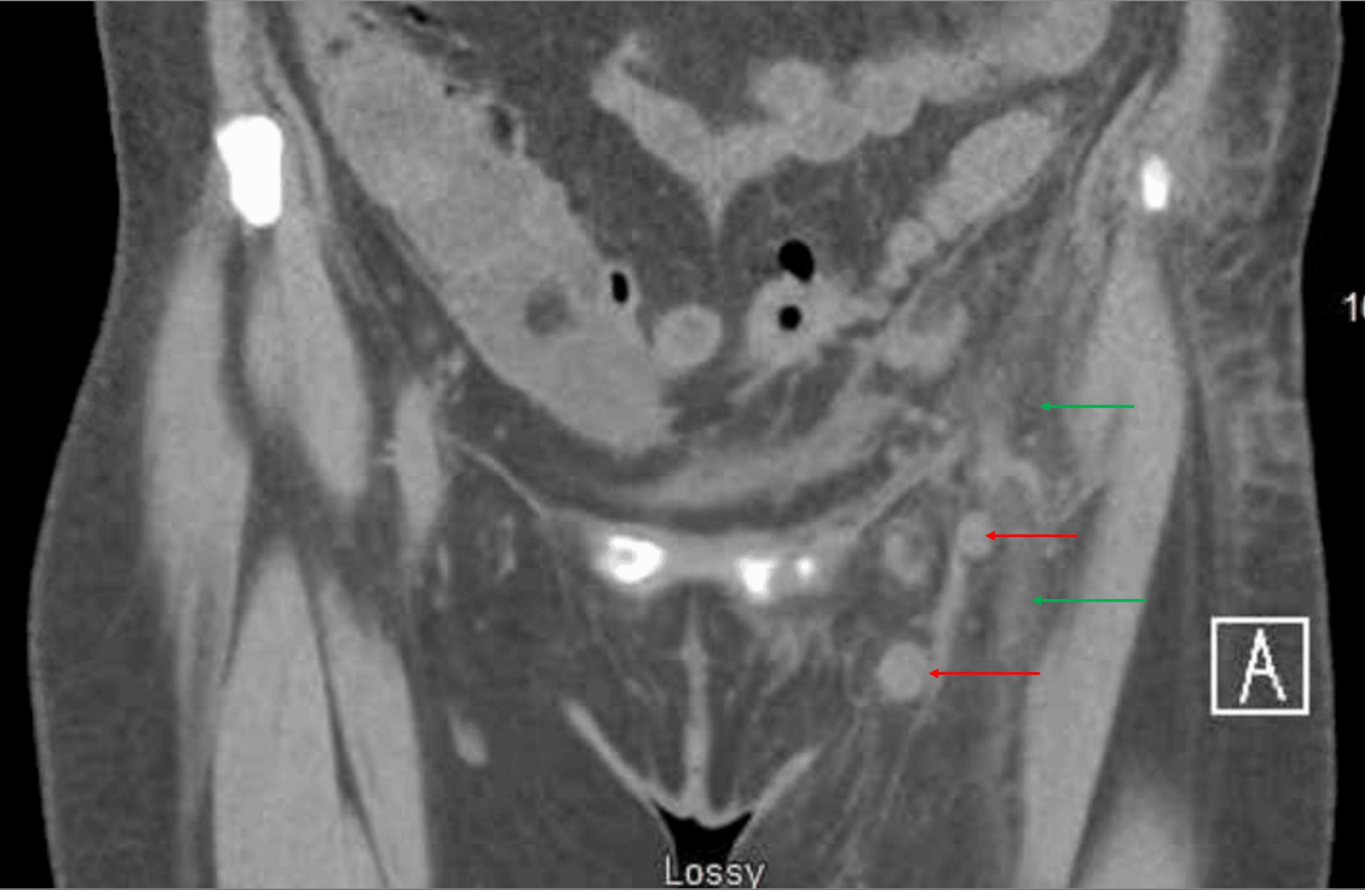
Computed tomography (CT) of the chest, abdomen, and pelvis revealed left groin extreme fat stranding (green arrow) extending into the pelvis with multiple enlarged left groin and iliac chain lymph nodes (red arrow).

On day two, the patient continued to be dyspneic, and computed tomography (CT) of the chest, abdomen, and pelvis revealed left groin extreme fat stranding extending into the pelvis with multiple enlarged left groin and iliac chain lymph nodes (Figure 1).

**Figure 2:**
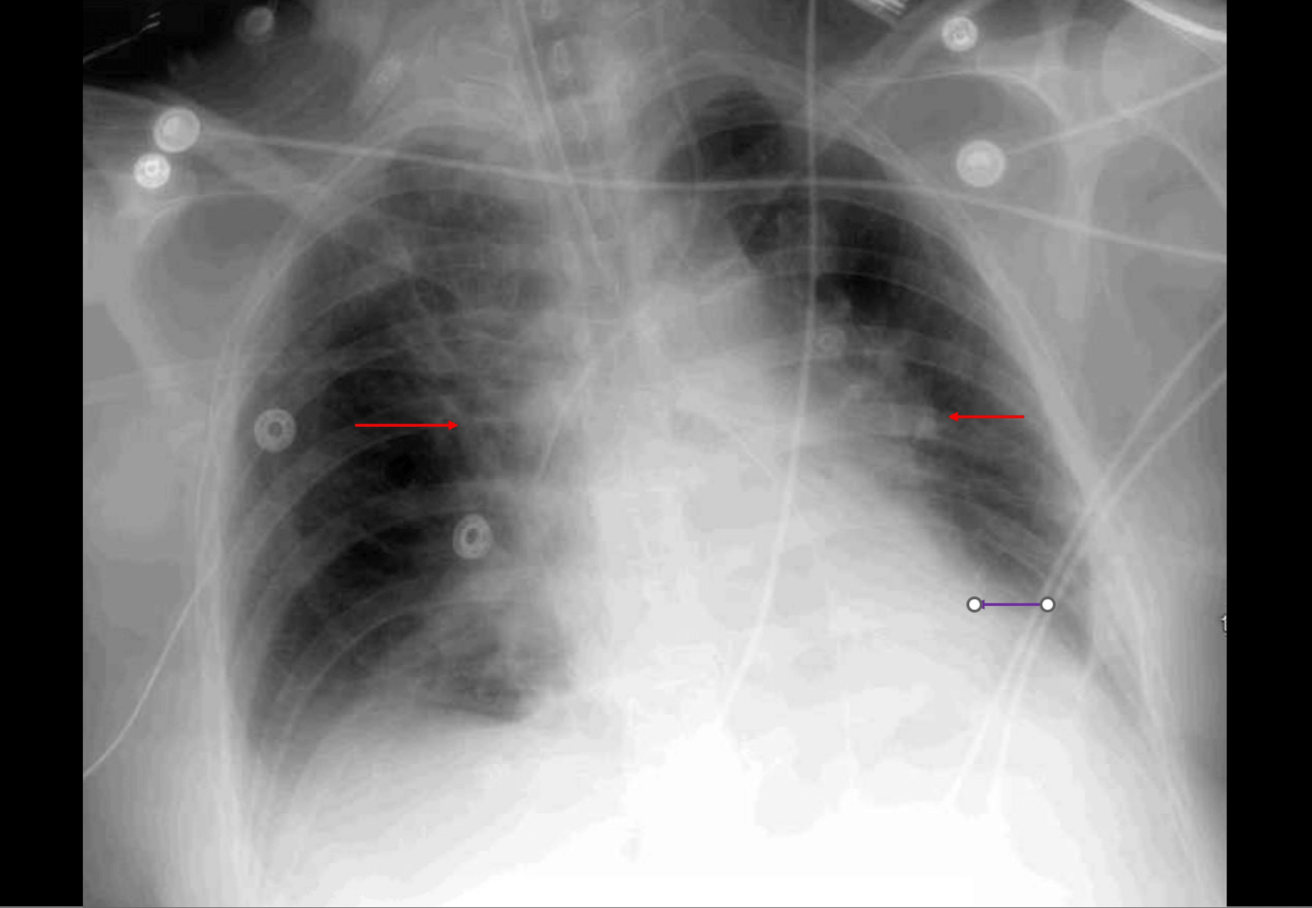
Chest X-ray done post-intubation revealed the appropriate position of the endotracheal tube, orogastric tube, and retrocardiac consolidation (purple arrow) with progressive perihilar pulmonary edema (red arrow).

General surgery was consulted, a bedside excisional debridement of the left groin region was performed, and tissue cultures were obtained, which resulted in mixed growth. Given leukopenia and thrombocytopenia, doxycycline was initiated to cover TBD. A ventilation-perfusion scan done due to elevated D-dimer and acute kidney injury (AKI) was negative for acute pulmonary embolism. The nephrology team consulted for AKI, and worsening metabolic acidosis recommended vigorous fluid hydration and strict monitoring of input and output with Foley catheter insertion with a concern for a need for hemodialysis in the next few days. On day three, she became hypotensive with cold limbs and hypoxic and developed atrial fibrillation with rapid ventricular response. She was initiated on amiodarone, vasopressors (norepinephrine and vasopressin), and stress dose steroids and transferred to the medical intensive care unit (MICU), where she was placed on mechanical ventilatory support on sedation after intubation. Chest X-ray done post-intubation revealed the appropriate position of the endotracheal tube, orogastric tube, and retrocardiac consolidation with progressive perihilar pulmonary edema (Figure 2).

Due to her persistent acidosis and AKI stage three, she was initiated on a bicarbonate drip and subsequently on temporary intermittent hemodialysis (HD) via a right femoral HD catheter. Peripheral smear revealed pancytopenia with megakaryoblasts, Döhle bodies, and toxic vacuolation with no schistocytes or pathogens. The stool workup was negative (Table 2) and serum ferritin returned was elevated (Table 1), suggesting HLH. Hemoncology team consult recommended considering etoposide and dexamethasone for HLH with no clinical improvement, and a stat soluble interleukin 2 receptor levels (sIL-2R) were sent. Piperacillin-tazobactam was changed to ertapenem.

**Table 2:** Other clinically significant laboratory results and imaging. ANA: antinuclear antibody, ANCA: antineutrophil cytoplasmic antibody, anti-GBM: antiglomerular basement membrane antibody, anti-PF4: IgG antibody against platelet factor 4 (PF4)/heparin complex

Laboratory test	Results
1) Autoimmune workup for acute kidney injury	Negative (ANA, ANCA, anti-GBM)
2) Acute viral hepatitis panel	Negative
3) Blood cultures at admission	Both sets were positive for F. tularensis
4) Blood cultures on day five post-admission	Both sets were positive for F. tularensis
5) Ehrlichiosis serology	Negative
6) Francisella tularensis serology at discharge	Positive for elevated IgM and IgG antibodies to F. tularensis
7) Heparin-induced thrombocytopenia panel	Negative (anti-PF4 reflexed to serotonin release assay)
8) Lyme disease serology	Negative
9) Left groin abscess culture	Mixed flora
10) Peripheral smear done at admission	Revealed leukopenia and thrombocytopenia, no blasts or schistocytes seen, Megakaryoblasts were seen, and positive for toxic vacuolation and Döhle bodies
11) Rickettsia serology	Negative
12) Stool pathogen panel	Negative
13) Stool ova and parasites	Negative
14) Stool culture	No growth
15) Arsenic, lead, and heavy metal toxicity	Negative
16) Ultrasound abdomen	Hepatomegaly (length > 20.6 cm) and spleenomegaly (length > 15 cm)
17) Transthoracic echocardiogram	Normal left ventricle systolic and diastolic function
18) CT chest	Bilateral moderate pleural effusions with adjacent compressive atelectasis and multifocal ground glass opacities inflammatory in nature
19) Positron emission tomography-CT	Negative for focal uptake, positive for multiple reactive left groin and iliac chain lymph nodes
20) Magnetic resonance imaging of the brain	Negative for any acute abnormality
21) CT head perfusion and angiogram of the head and neck	Negative for any acute intracranial and vascular abnormality

On day four, labs revealed elevated sIL-2R and pancytopenia (Table 1). Due to no clinical improvement, she was started on the first dose of IV etoposide and dexamethasone 20 mg as malignancy-induced HLH was suspected. Due to a bad sample, the fibrinogen level could not be determined. Fluconazole and acyclovir were added, and ertapenem was switched to imipenem for pseudomonas coverage. On day five, intermittent HD was continued along with vasopressors, and the bicarbonate drip was stopped. Repeat blood cultures were sent. Oozing from the peripheral, central, and arterial lines seen on day four stopped, and the patient was weaned off the vasopressors. On day six, pancytopenia was persistent, and blood cultures obtained at admission returned positive for *F. tularensis*. The sample was forwarded to the state lab for confirmation. Vancomycin was stopped, doxycycline was continued, and gentamicin was initiated after the HD session. A second dose of etoposide was administered while waiting for blood culture confirmation. She also received two units of packed red blood cells (PRBCs) and platelets. By day eight, serum ferritin, liver, and kidney functions improved, and pancytopenia worsened. TBD serologies returned negative (Table 2). Blood cultures obtained on day five returned positive for *F. tularensis*. The state lab confirmed the positive blood cultures, and the patient was transferred from the OSH to a tertiary academic institution (University of Missouri Healthcare, Columbia) MICU, for a higher level of care.

**Figure 3:**
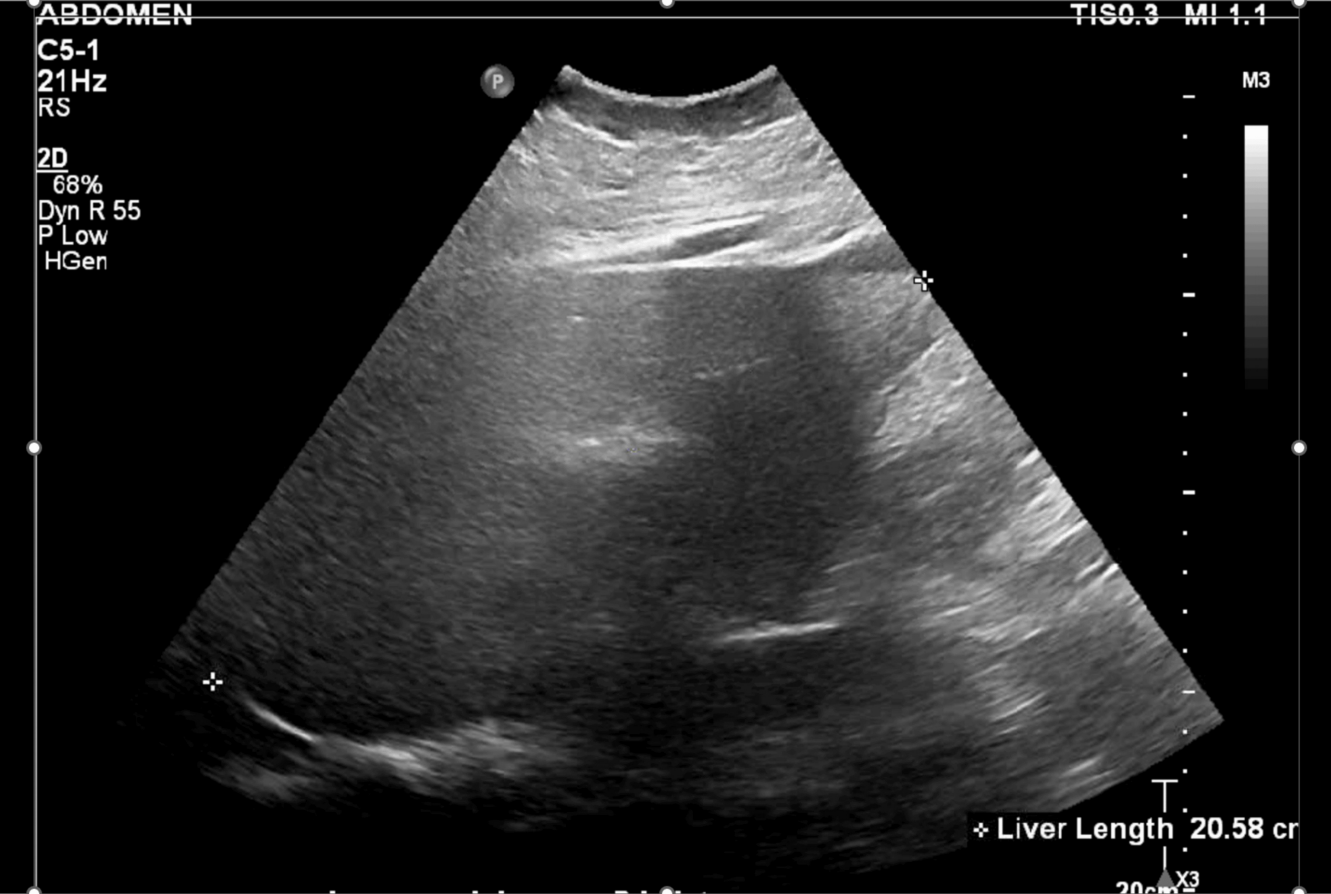
Ultrasound abdomen revealed hepatomegaly (>20 cm).

**Figure 4:**
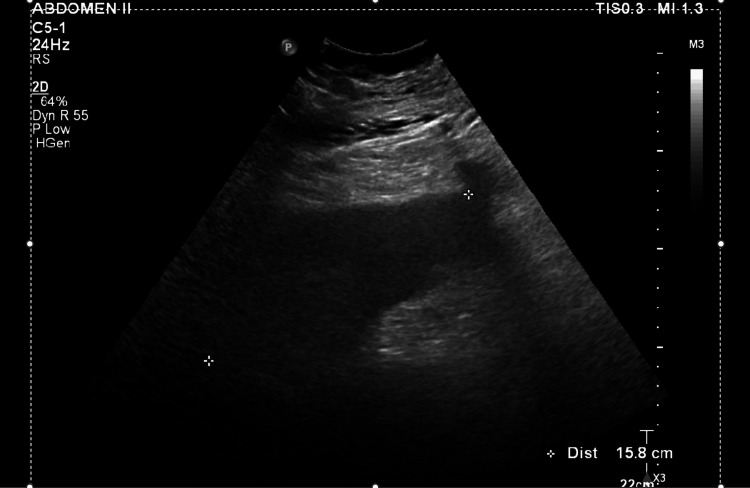
Ultrasound abdomen revealed spleenomegaly (>15 cm).

At our MICU, the patient was continued on mechanical ventilatory support, antimicrobials (imipenem was changed to meropenem), and dexamethasone. Her arial fibrillation was well controlled on amiodarone. Serum triglycerides and fibrinogen obtained after transfer were abnormal (Table 1). A natural killer (NK) cell functional assay returned low (Table 1). Ultrasound of the abdomen revealed hepatosplenomegaly (Figures 3, 4) (Table 2).

**Figure 5:**
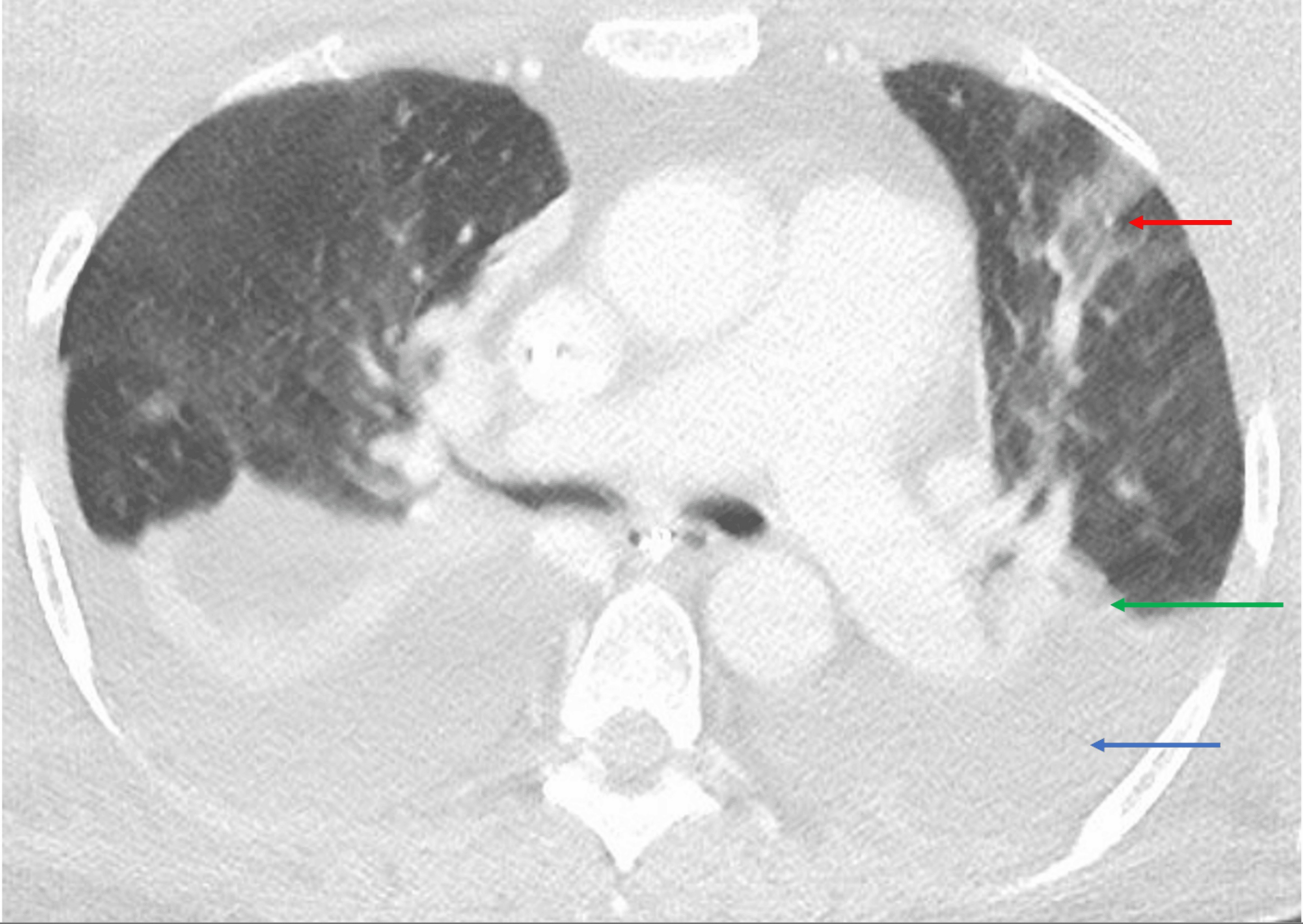
CT of the chest revealed bilateral moderate pleural effusions (blue arrow) with adjacent compressive atelectasis (green arrow) and inflammatory multifocal ground-glass opacities (red arrow).

CT of the chest revealed bilateral moderate pleural effusions with adjacent compressive atelectasis and inflammatory multifocal ground-glass opacities (Figure 5).

Due to transient confusion, brain imaging done was within normal limits (Table 2). The hematology team recommended transfusing irradiated PRBC and platelets to maintain target hemoglobin >7.5 gm/dl and a platelet count >50,000/mL. The hematology team agreed with the diagnosis of HLH disease due to acute tularemia with septic shock and recommended no etoposide and gradual tapering of dexamethasone. She was positive for seven of the eight HLH 2004 criteria except for the histopath biopsy confirmation (Table 3) [4].

**Table 3:** Hemophagocytic lymphohistiocytosis (HLH)-2004 diagnostic criteria. The diagnosis of HLH can be established if criterion 1 or 2 is fulfilled. Source: [4]

Criterion	HLH Diagnostic Criteria
Criterion 1	A molecular diagnosis consistent with HLH
Criterion 2 (five of the following eight criteria)	1) Fever. 2) Splenomegaly. 3)Cytopenias (affecting ≥2 of 3 lineages in the peripheral blood): hemoglobin < 90 g/L (hemoglobin < 100 g/L in infants < 4 wk); platelets <100 ×109/L neutrophils <1.0 ×109/L. 4) Hypertriglyceridemia and/or hypofibrinogenemia: fasting triglycerides ≥ 3.0 mmol/L (i.e., ≥ 265 mg/dL); fibrinogen ≤ 1.5 g/L. 5) Hemophagocytosis in the bone marrow or spleen or lymph nodes. No evidence of malignancy. 6) Low or no NK cell activity (according to local laboratory reference). 7) Ferritin ≥ 500 µg/L. 8) sCD25 (i.e., soluble IL-2 receptor) ≥ 2400 U/mL.
Supportive evidence	1) Cerebral symptoms with moderate pleocytosis and/or elevated protein. 2) Elevated transaminases. 3) Elevated bilirubin. 4) Elevated LDH.

The HSCORE was 260 with a >99% probability of HLH. The heparin-induced thrombocytopenia panel returned negative, as did the autoimmune workup for AKI (Table 2). Over the next week, the intermittent HD sessions were gradually stopped as the patient entered the polyuric phase of acute tubular necrosis. The infectious disease (ID) team recommended continuing the gentamicin plus meropenem, and doxycycline was stopped. Intermittent PRBC and platelets were administered as needed. She was then extubated to room air approximately seven days prior to discharge. Due to pancytopenia, the ID team recommended switching fluconazole to posaconazole. Her pancytopenia resolved during the last week of hospitalization, and antimicrobials (meropenem, acyclovir, and posaconazole) were stopped. She completed a 10-day course of gentamicin. Dexamethasone was switched to prednisone 50 mg daily with a gradual taper over the next four weeks. A repeat NK cell functional assay returned normal, and an *F. tularensis *serology returned positive (Tables 1, 2). She improved clinically along with her mentation and was resumed on an oral diet. She was eventually discharged to an acute rehab facility for a few weeks due to deconditioning on a prednisone taper and oral amiodarone. No residual deficits on examination were noted when she followed up with her primary care physician five weeks after discharge.

## Discussion

Tularemia is a rare zoonotic disease with an incidence of 0.05 per 100,000 individuals in the USA [5]. Multiple strains of *F. tularensis* exist, with diverse virulence and clinical presentation severity during spring to early fall. Transmission vectors responsible are ticks and deerflies, which acquire the bacteria from an infected muskrat or cottontail rabbit. Ticks accounting for the majority of cases are the lone star (*Amblyomma americanum*), dog (*Dermacentor variabilis*), and wood tick (*Dermacentor andersoni*) in the USA [4]. Acute tularemia causing HLH has not been reported yet. Two extensive nationwide studies revealed infection as the most common cause of HLH [2,3]. In both, viral causes outnumbered the bacterial causes of HLH. Among the infectious causes, Epstein-Barr virus (EBV) was the most common cause, accounting for more than 50% of all infectious causes. TBD-causing infectious HLH in both these studies was almost negligible (<1%).

HLH is a rare and often fatal disease resulting from an aberrant hyperimmune response. It is classified into HLH syndrome and HLH disease. Primary causes are mutations in genes accountable for NK and cytotoxic T cell lymphocyte (CTL) cytotoxic activity, often seen in infants and younger children. Secondary causes include infections, autoimmune, autoinflammatory, malignancy, acquired immune defects, and medications [6]. Mechanisms implicated in secondary HLH are dual. The first step is immune system activation due to stimulation of the toll-like receptors on the NK cell surface by specific components of viruses, bacteria, and fungal organisms. The second step is an abject failure of NK cells and CTLs to eradicate activated macrophages, resulting in a vicious cycle of persistent activation of CD8+ T cells and macrophages. The dual mechanisms result in a cytokine storm responsible for multiorgan failure and high mortality associated with HLH [1,7,8]. HLH often presents as an acute or subacute illness with multiorgan involvement, making the diagnosis a challenge due to nonspecific symptoms but often with a triad of fever, bicytopenia, and splenomegaly [4]. Unfortunately, no single pathognomic clinical sign or laboratory abnormality is sensitive or specific to make a diagnosis. HLH diagnosis is established by fulfilling the HLH-2004 diagnostic criteria (Table 3) concomitantly with clinical reasoning and patient presentation [9]. The HLH-2004 criteria have not been validated in adults with secondary HLH, and often, an alternative HSCORE is used with a score >250, indicating a high HLH probability [10]. The HSCORE performance is better when utilized at an initial presentation before worsening [11]. In adults, a sensitive indicator is sIL-2R, with levels <2,400 U/mL ruling out HLH and levels >10,000 U/mL ruling in HLH [12]. A distinct cytokine profile (severe elevation in interferon-γ and IL-10 with a modest elevation in IL-6) in children helps differentiate HLH from infection [13]. Recognizing HLH promptly and identifying the underlying cause is essential for timely, accurate therapy.

HLH due to infection is seen more frequently in immunocompetent than immunosuppressed patients. Most bacterial causes of HLH are due to intracellular organisms such as *Rickettsia* [14]. To date, 98 cases of infectious HLH due to TBD have been reported, and the exact incidence is unknown, with maximum reports from the USA [15]. *Ehrlichia* was the most common tick-borne pathogen, followed by *Rickettsia* and *Anaplasma phagocytophilum*. *F. tularensis-induced* HLH has not been reported yet. Incidence in males was almost twice that seen in females. Almost half (43.9%) of TBD HLH cases were treated with antimicrobials, and 88.4% recovered completely without immunosuppression treatment. A combination of antimicrobial and immunosuppressive treatment was utilized in 52% of patients, and 4.1% were treated with immunosuppressive therapy alone. Immunosuppressive therapy alone failed with no survival. Steroids were the most commonly used immunosuppressive therapy. Of patients who died while on antimicrobial therapy, only 30.8% received appropriate early, timely antimicrobials, whereas late, inappropriate antimicrobials were observed in 69.2% of patients. The mortality rate in TBD HLH was 16.3% higher than TBD mortality (10%) but lower than the overall mortality rate of 41% in HLH. The survival rate of TBD HLH cases was higher in comparison to other secondary causes of HLH. *Ehrlichia* spp. had the highest mortality among TBD HLH, followed by *Rickettsia* and *Anaplasma* *phagocytophilum*. Adequate, appropriate, and timely antimicrobial therapy resulted in a better survival rate [15].

Our patient resided in a Southern Missouri rural area farmhouse with gardens visited by rabbits and had a tick bite a week before presentation, followed by left groin swelling. She denied recent visits to parks or consuming contaminated agricultural products and water. She presented with a left groin glandular lesion and septic shock. She fulfilled seven of the eight HLH-2004 criteria and had an HSCORE > 260. Timely initiation of antimicrobial therapy on day two, followed by an immunosuppressive regimen, resulted in a better outcome. She completed a 10-day course of gentamicin and gradually recovered to her baseline. Based on our medical literature review via PubMed, this is the first reported case of acute HLH due to tularemia.

## Conclusions

Given that HLH is rare, it is associated with a high mortality rate if there is a failure in prompt diagnosis and treatment. As tularemia is endemic in Missouri, it should be considered an etiologic agent for HLH in this area along with ehrlichiosis. Further research needs to identify a cytokine profile in adults that can differentiate infection from HLH. As both therapeutic regimens, antimicrobials alone or in combination with immunosuppressives, were used, it is unclear which regimen is superior. As clinically evident in our case, timely recognition followed by an appropriate, early antimicrobial treatment with immunosuppressives is essential in improving patient outcomes. Future research is needed to identify the most effective and safe immune-modulating therapeutic regimen for infectious causes of HLH.

## References

[1] Janka GE, Lehmberg K (2014). Hemophagocytic syndromes--an update. Blood Rev.

[2] Ishii E, Ohga S, Imashuku S (2007). Nationwide survey of hemophagocytic lymphohistiocytosis in Japan. Int J Hematol.

[3] Birndt S, Schenk T, Heinevetter B (2020). Hemophagocytic lymphohistiocytosis in adults: collaborative analysis of 137 cases of a nationwide German registry. J Cancer Res Clin Oncol.

[4] La Rosée P, Horne A, Hines M (2019). Recommendations for the management of hemophagocytic lymphohistiocytosis in adults. Blood.

[5] Auwaerter P, Penn R (2020). 227. Francisella tularensis (Tularemia). Mandell, Douglas, and Bennett's Principles and Practice of Infectious Diseases (9th ed).

[6] Janka GE (2012). Familial and acquired hemophagocytic lymphohistiocytosis. Annu Rev Med.

[7] Takeuchi O, Akira S (2010). Pattern recognition receptors and inflammation. Cell.

[8] Kaçar AG, Celkan TT (2022). Hemophagocytic lymphohistiocytosis. Balkan Med J.

[9] Henter JI, Horne A, Aricó M (2007). HLH-2004: diagnostic and therapeutic guidelines for hemophagocytic lymphohistiocytosis. Pediatr Blood Cancer.

[10] Fardet L, Galicier L, Lambotte O (2014). Development and validation of the HScore, a score for the diagnosis of reactive hemophagocytic syndrome. Arthritis Rheumatol.

[11] Debaugnies F, Mahadeb B, Ferster A, Meuleman N, Rozen L, Demulder A, Corazza F (2016). Performances of the H-score for diagnosis of hemophagocytic lymphohistiocytosis in adult and pediatric patients. Am J Clin Pathol.

[12] Hayden A, Lin M, Park S (2017). Soluble interleukin-2 receptor is a sensitive diagnostic test in adult HLH. Blood Adv.

[13] Xu XJ, Tang YM, Song H (2012). Diagnostic accuracy of a specific cytokine pattern in hemophagocytic lymphohistiocytosis in children. J Pediatr.

[14] Cascio A, Pernice LM, Barberi G (2012). Secondary hemophagocytic lymphohistiocytosis in zoonoses. A systematic review. Eur Rev Med Pharmacol Sci.

[15] Jevtic D, da Silva MD, Haylock AB (2024). Hemophagocytic lymphohistiocytosis (HLH) in patients with tick-borne illness: a scoping review of 98 cases. Infect Dis Rep.

